# Annotations

**Published:** 1906-02-24

**Authors:** 


### ANNOTATIONS.

### The Children of the Doctor.

BISHOP WELLDON, in the current number of the "Nineteenth Century" has contributed an interesting article on "The Children of the Clergy." He has analysed the parentage of distinguished men and women whose names appear in the "Dictionary of National Biography," with the result that while he has been able to collect 1,270 eminent or prominent children of the clergy since the Reformation, "the children of lawyers and doctors who have attained eminence or prominence in all English history have, upon a calculation as accurate as it has proved possible to make, been 510 and 350. Bishop Welldon believes that the sons and daughters of the clergy have rendered greater service to the State than any other class, and he evidently makes little count of the medical profession in producing a "distinguished" progeny. Striking as these figures may be at the first glance, they prove but little, and apparently no attempt has been made to estimate the relative proportion of the classes compared or to allow for the social and educational advantages rigorously safeguarded by the clergy in bygone days. We are all acquainted with the analysis of the late Sir James Paget, in which he gave statistical expression to the chances of "success" likely to be attained in the medical world, and Dr. Squire Sprigge's more recent returns are of considerable value. It now remains for someone with discernment and discriminating care to ascertain whether the number of children of distinction is really proportionately so much smaller in the medical than in the clerical profession as Bishop Welldon's figures have suggested. Should such, indeed, prove to be the case, it is desirable that the etiological factors be thoroughly ascertained.

### Parliament and Juvenile Smoking.

ANOTHER attempt is to be made this Session to put down juvenile smoking by Act of Parliament. The measure is on the same lines as that which was brought in last year, founded upon the recommendations of the Physical Deterioration Committee of 1904, and the principal clause aims at the prohibition of the sale of tobacco and cigarettes to any child under the age of sixteen. Moreover, any child under the same age found smoking any kind of tobacco will be liable, if the Bill passes, to punishment on conviction. Thus, it is proposed to strike at both parties, and it is stated that the Bill is warmly approved by several distinguished soldiers as well as by numerous eminent medical men. The promoters anticipate that if in the ballot they can secure a favourable place on the Order Book there will be no difficulty in getting the Bill added to the statute book. We are afraid that they are unduly sanguine. Most sensible people are by this time quite convinced that tobacco smoking in any form by children is highly detrimental to health and morals, and ought by some means to be prevented. The new House of Commons might be easily persuaded to sanction a proposal to inflict, under due precautions, a substantial fine upon shopkeepers who supply the cause of the mischief. But it remains to be seen whether it will assent to the punishment of children who smoke. It would, of course, refuse to enact that a child under sixteen who is caught puffing a cigarette shall be committed to prison, even on a repetition of the offence, for that remedy would be worse than the disease. One alternative appears to be the employment of the birch in some shape or other. We do not say that this would not be salutary, and it might be effective. But we shall be surprised if it commends itself to a Parliament which seems more likely to err on the side of sentiment than on that of stringency. A more reasonable alternative would be to fine the parents of the offenders for the disobedience of their offspring to the law.

### Black Fogs.

THE "London particular" fog is so much an institution of the metropolis that imagination staggers to contemplate a London from which London fog has been permanently banished: we say "London fog" advisedly, because this variety is a triumph of art, not nature, and is as preventible, theoretically, as is (theoretically again) tuberculosis, or typhoid fever, or syphilis, or a variety of other scourges whose prevention we are cilivised enough to know but not yet civilised enough to practise. On another page will be found a brief abstract of a paper on this subject delivered by Sir Oliver Lodge, in which he outlines the wise, economical, and obvious method of avoiding the typical London fog, though, of course, its basis, the white fog, will always be with us. It is not to be denied that the white fog is nuisance enough as a disturber of traffic, but it does not harry the nostrils with sulphurous fumes and bring tears to the eyes; it does not turn day into night, nor deposit a veneer of lamp-black upon every exposed surface. It may be that the suspended products of faulty combustion which constitute the black fog are not actively harmful to the human economy, though the effect of such a fog upon bronchitic subjects leaves even this hypothesis in doubt; but there can be no question of the damaging nature of fog upon fabrics and furniture. London fog is rich in sulphurous acid, which is rapidly oxidised into oil of vitriol, and may be relied upon in this latter condition to complete the work of destruction well begun by the greasy nature of its vehicle. Certain optimists have taken comfort from the reflection that London fogs are antiseptic and bactericidal, but even this consolation is derided by Sir Oliver Lodge. The harms and distresses caused by fogs are well known and commonplace, although, as Sir Oliver trenchantly puts it, "they can hardly be repeated too frequently so long as the barbarous combustion of crude coal in a savage and unorganised manner is permitted in the midst of the semi-civilisation we have so far attained."

